# Assessment of Control Tissue for Gene and Protein Expression Studies: A Comparison of Three Alternative Lung Sources

**DOI:** 10.1100/2012/523840

**Published:** 2012-04-19

**Authors:** Margaret R. Passmore, Maria Nataatmadja, John F. Fraser

**Affiliations:** ^1^Critical Care Research Group, University of Queensland, Prince Charles Hospital, Brisbane, Australia; ^2^Department of Medicine, University of Queensland, Prince Charles Hospital, Brisbane, Australia

## Abstract

The use of an appropriate control group in human research is essential in investigating the level of a pathological disorder. This study aimed to compare three alternative sources of control lung tissue and to determine their suitability for gene and protein expression studies. Gene and protein expression levels of the vascular endothelial growth factor (VEGF) and gelatinase families and their receptors were measured using real-time reverse transcription polymerase chain reaction (RT-PCR) and immunohistochemistry. The gene expression levels of VEGFA, placental growth factor (PGF), and their receptors, fms-related tyrosine kinase 1 (FLT1), and kinase insert domain receptor (KDR) as well as matrix metalloproteinase-2 (MMP-2) and the inhibitors, tissue inhibitor of matrix metalloproteinase-1 (TIMP-1) and TIMP-2 were significantly higher in lung cancer resections. The gene expression level of MMP-9 was significantly lower in the corresponding samples. Altered protein expression was also detected, depending on the area assessed. The results of this study show that none of the three control groups studied are completely suitable for gene and protein studies associated with the VEGF and gelatinase families, highlighting the need for researchers to be selective in which controls they opt for.

## 1. Introduction

In order to determine the presence and level of a pathological disorder, an appropriate control is essential to use as a standard baseline of the normal condition. In animal experiments, controls are relatively easy to obtain, but in many human studies this can be problematic especially if the experiments involve vital organs such as lung, heart, and kidney. Due to these short comings, researchers often have to find alternative sources of normal tissue. The decision to use control tissue from a “nonnormal” source is not ideal and should only be considered as a last resort. If researchers have to use this source of tissue, it is critical that they choose an appropriate group. Thus, the aim of this study was to compare several alternative sources of control lung and to determine their suitability for gene and protein expression studies.

 A review of the published literature shows that specimens from lung cancer resections are used frequently as controls in respiratory research in a wide range of conditions including bronchiolitis obliterans syndrome (BOS), chronic obstructive pulmonary disease (COPD), bacterial infection, and idiopathic pulmonary fibrosis [1–4]. Lung cancer resections are readily available and easy to obtain; however, this source of control tissue potentially has circulating mediators released due to the presence of lung cancer.

While using fresh or frozen tissue is ideal for RNA quality, this is not always possible. Improvements in RNA extraction technology have allowed us to extract RNA from formalin-fixed paraffin-embedded tissue, opening up the possibility of using archived samples. Having access to multiple, alternative sources of control tissue will allow investigators to choose the best representative for their study condition. We aimed to compare and validate three alternative sources of control lung tissue including neoplastic tissue, biopsies from stable post-lung-transplantation patients, and archived formalin-fixed tissue from pneumothorax patients.

 We have selected specific lung injury biomarkers relating to the VEGF family including vascular endothelial growth factor A (VEGFA), placental growth factor (PGF), and their receptors, fms-related tyrosine kinase 1 (FLT1), kinase insert domain receptor (KDR), as well as the gelatinases, matrix metalloproteinase-2 (MMP-2) and -9 (MMP-9) and their inhibitors, tissue inhibitor of matrix metalloproteinase-1 (TIMP-1) and -2 (TIMP-2).

VEGF is expressed in multiple cell types in lung including muscle, epithelial lining, macrophages, and endothelial cells [5, 6]. It is highly expressed during lung development [7], and its upregulation during maturity is associated with the presence of lung disease such as oedema [8], emphysema [9], and the development of PGD after transplantation [10]. Increased VEGF expression has also been reported in lung cancer tumours in association with angiogenesis [11] and activated macrophages [12]. PGF is a member of the VEGF family and is expressed in the human placenta under normal conditions [13] but is upregulated in certain pathological conditions such as wound healing, pulmonary emphysema, and tumour formation [9, 14, 15]. The receptors FLT1 and KDR mediate the effects of both VEGF and PGF and have been shown to be simultaneously upregulated in the presence of lung disease [7, 8, 16]. MMP-2 and MMP-9 have been implicated following lung transplant remodelling and injury [17] as well as having pathological roles in inflammation, cancer, and cardiovascular disease [18, 19]. MMPs are also involved in many normal processes including embryonic development, morphogenesis and tissue remodeling, and wound healing [20, 21]. TIMP-1 and -2 are important enzymes in regulating the balance of MMPs, and a disparity can lead to pathological remodelling.

These biomarkers are important for detection of early pathological changes in lung structure and function including the presence of primary graft dysfunction (PGD) and BOS in lung transplantation.

The aim of this study, therefore, was to determine the suitability of three alternative sources of control tissue derived from fresh as well as formalin-fixed tissue and to assess the basal gene and protein expression levels for these specific lung injury biomarkers.

## 2. Methods

### 2.1. Patients and Sample Collection

#### 2.1.1. Lung Cancer Resection

Samples were obtained from lung cancer patients undergoing lung resection (*n* = 29) where sections were screened to exclude those containing local tumour, severe emphysema, fibrosis, and vascular invasion. Samples were only collected from patients with a primary tumour, with no lymph node involvement or distant metastasis as detailed in the pathologist's histopathology report. These samples included 4 nonsmokers.

#### 2.1.2. Stable Post-Lung-Transplant Biopsy

Biopsies were obtained from bilateral lung transplantation patients where 38 biopsies were collected during diagnostic and surveillance bronchoscopies. Samples ranged from 1 week to over 2 years following transplantation, and, of these, 26 were classified as stable.

#### 2.1.3. Pneumothorax Archived Tissue

The final group were patients admitted with pneumothorax (*n* = 15) where samples were obtained retrospectively from archived tissue blocks, less than 2 years old. These samples had previously been fixed in formalin and embedded in paraffin.

All experimental procedures were conducted with the approval of The Prince Charles Hospital Research and Ethics Committee (EC2639) with written informed consent from the subjects. Lung cancer resection samples and lung transplantation biopsies were collected in RNA*later *(Ambion, Calif, USA) for RNA extraction and 10% buffered formalin for histological analysis.

### 2.2. Isolation of RNA and cDNA Synthesis

Total RNA was isolated from biopsies and lung resections using Trizol (Ambion, Calif, USA) and samples purified with the RNeasy Mini Kit (Qiagen, Australia). RNA was extracted from pneumothorax samples using the RecoverAll Total Nucleic Acid Isolation Kit (Ambion, Calif, USA) for formaldehyde- or paraformaldehyde-fixed, paraffin-embedded tissues. All samples were DNase treated (Ambion, Calif, USA) and subsequently analysed on an Agilent Bioanalyzer (Agilent Technologies, Australia) to determine RNA concentration and quality. First strand cDNA was synthesized from 400 ng of RNA using random primer and AMV Reverse Transcriptase (Roche, Basel, Switzerland).

### 2.3. Real-Time PCR

Primers were purchased from Applied Biosystems (Table 1). Real-time quantitative RT-PCR was performed using a Rotor-Gene 6000 real-time rotary analyzer (Corbett Research, Australia) with Universal PCR Master Mix containing UNG AmpErase (Applied Biosystems, USA). The cycling conditions were as follows: cDNA was denatured at 95°C for 10 min, followed by 40 cycles of 95°C for 15 s and 60°C for 60 s. A no-template and reverse transcription negative control was included for each primer set. Threshold cycle (Ct) values from the Rotor-Gene software version 1.7 (Corbett Research, Australia) were exported to Microsoft Excel for further analysis. All measurements were performed in triplicate for each gene, and samples were quantified from standard curves, using serial dilutions of a cDNA pool of all lung samples. GeNorm 3.4 software was used to determine the most stable reference genes, and all genes of interest were subsequently normalised to GUSB, HGPRT, and B2M.

### 2.4. Immunohistochemical Staining

Lung tissue was fixed in formalin for at least 48 hrs, and processed, and embedded in paraffin, and 4 *μ*m serial sections were cut. Prior to the application of primary antibodies, the sections were subjected to antigen retrieval by heating at 95°C for 20 mins in citrate buffer followed by blocking endogenous peroxidase by incubating them in 3% H_2_O_2_ for 5 min. Nonspecific binding was blocked by incubating them in 10% normal horse serum for 1 hr (Vector Lab, Calif, USA). Primary antibodies against VEGF, PGF (1 : 200), FLT1, KDR, TIMP-2 (1 : 200), MMP-2, TIMP-1 (1 : 100), and MMP-9 (1 : 40) were purchased from Santa Cruz (Calif, USA). Antibodies were applied and then incubated overnight at 4°C. Macrophages were identified using CD68 (1 : 200; Dako, Calif, USA). The Vector universal ABC secondary antibody kit (Vector Lab, Calif, USA) was used to link the primary antibody to the chromogen and incubated for 1 hr. A positive reaction was detected with 3,3′-diaminobenzidine tetrahydrochloride (Sigma-Aldrich, Munich, Germany) which produces a brown colour at the site of the reaction.

### 2.5. Immunoreactivity Scoring

Images were photographed at a magnification of 250x and visualised using the AxioVision 4.7 Image Analysis system. Semiquantitative analysis was performed by an independent investigator blinded to the slide identity. The staining intensity was assessed and scoring recorded in the bronchiole and alveolar lining of the lung tissue as well as in the inflammatory cells including macrophages and polymorphonuclear leucocytes (PMNs). The intensity of positive staining was scored as negligible (0), mild (1), moderate (2), and strong (3).

### 2.6. Statistical Analysis

Comparison between the three control groups was determined using a one-way ANOVA with Tukey's multiple comparison test for gene expression studies, where statistical significance was assumed when *P* < 0.05.

Gene and protein expression was considered at a basal level when all three groups showed similar expression levels. If one group differed in expression, this group was excluded as a control source for the measurement of that particular biomarker.

## 3. Results

### 3.1. Study Population

Patient demographics can be seen in Table 2. Lung cancer patients had a higher age compared to either lung transplant or pneumothorax patients.

### 3.2. Gene Expression Levels

**Figure 1:**
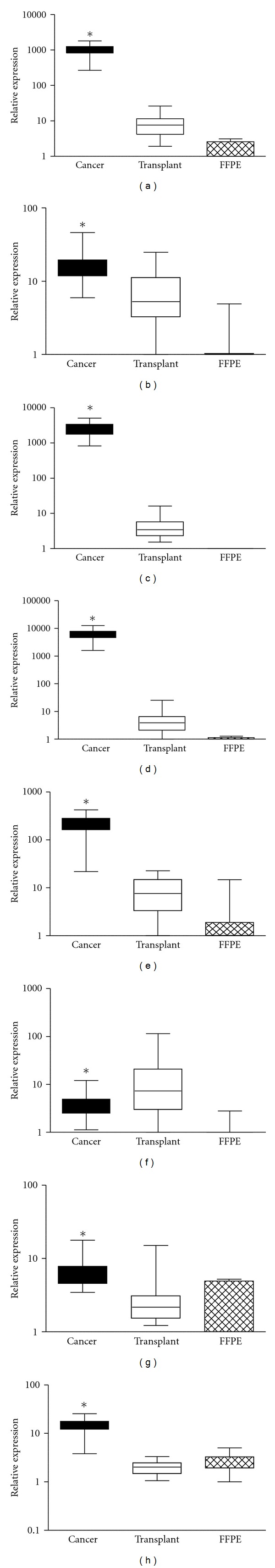
Quantitative real-time PCR analysis of VEGFA (a), PGF (b), FLT1 (c), KDR (d), MMP-2 (e), MMP-9 (f), TIMP-1 (g), and TIMP-2 (h). Gene expression levels were increased for VEGFA, PGF, FLT1, KDR, MMP-2, TIMP-1, and TIMP-2 in lung cancer (*n* = 29) compared to formalin-fixed paraffin-embedded (FFPE) tissue (*n* = 7) and stable lung transplant biopsies (*n* = 26). The levels of MMP-9 were significantly decreased. Results are expressed as mean ± SD. Asterisks indicate *P* < 0.05.

Biopsies from post-lung-transplant patients were grouped into the stable category (*n* = 26) by an independent assessor. Tissue from lung cancer resections (*n* = 29) and pneumothorax patients (*n* = 15) were processed in parallel. Intact RNA was successfully extracted from all lung cancer resection and lung transplant biopsy tissue; however, assessment of RNA integrity for the formalin-fixed paraffin-embedded pneumothorax tissue revealed intact RNA for only seven samples. The gene expression level of VEGFA, PGF, and their receptors FLT1 and KDR as well as MMP-2 and the inhibitors TIMP-1 and TIMP-2 was significantly higher in lung cancer specimens compared to pneumothorax samples and biopsies from lung transplant patients (*P* < 0.001; Figure 1). The gene expression level of MMP-9, however, was lower in lung cancer specimens compared to the other two groups.

Analysis of lung cancer resection tissue from the four nonsmoking patients compared to the smoking group showed there was no significant difference between the two groups in the expression of any of the genes examined (data not shown).

### 3.3. Histological Evaluation

**Figure 2:**
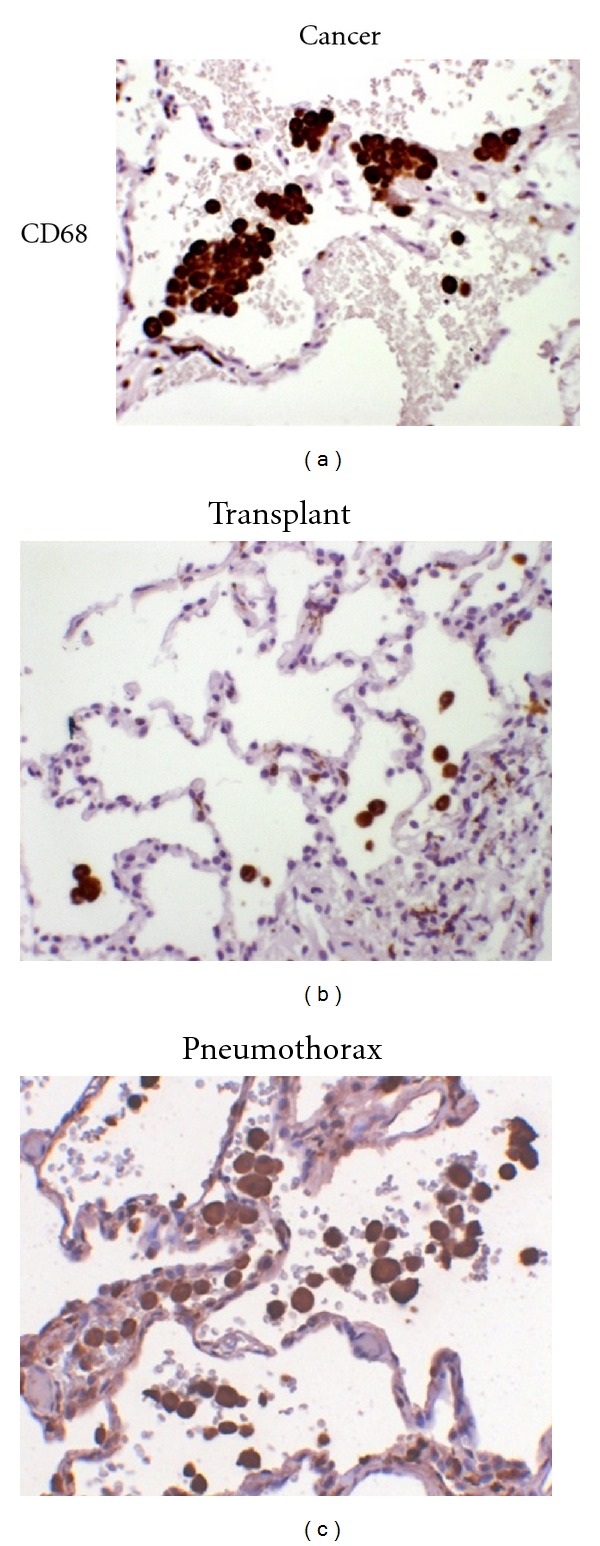
Immunohistochemical staining for CD68. The distribution of CD68+ macrophages in lung cancer resection (a), transplant biopsy (b), and pneumothorax (c). Magnification 250x.

When determining pathological changes in lung function, the bronchial and alveolar lining and inflammatory cell infiltrate are considered the most important area of assessment. Our study found abundant macrophages in the lung cancer and pneumothorax sections with clusters situated mostly in the alveolar spaces throughout the entire section. Some macrophages were also found in the transplant biopsy but in markedly lower numbers (Figures 2(a), 2(b), and 2(c)). PMNs were also found abundantly in lung cancer though the amount and distribution varied from sample to sample. Only occasional PMNs were found in pneumothorax and transplant biopsy samples. Due to this variability, scoring was not done in PMNs. Protein expression was, therefore, assessed in the bronchial and alveolar lining as well as in the macrophage infiltrate.

#### 3.3.1. Bronchiole

**Figure 3:**
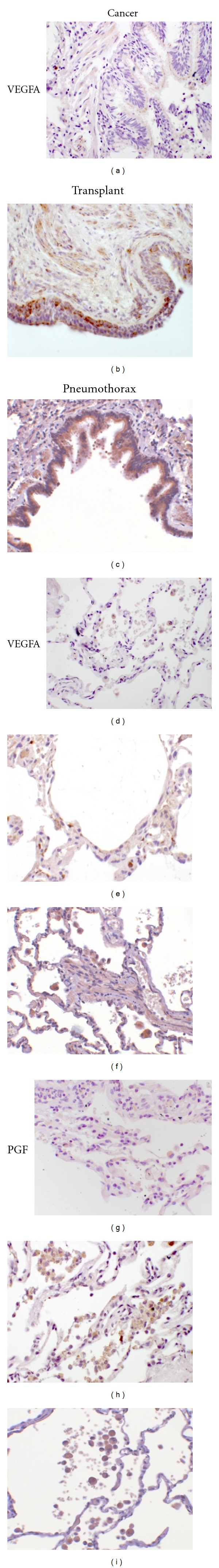
Representative immunohistochemical staining in the bronchial and alveolar lining and macrophages. The level of VEGFA expression was diminished in the bronchial lining of cancer resection (a) compared to transplant biopsy (b) and pneumothorax (c). The alveolar lining shows elevated levels of VEGFA in pneumothorax (f) compared to cancer resection (d) and transplant biopsy (e). The level of PGF expression was diminished in macrophage infiltrate in lung cancer (g) compared to transplant biopsy (h) and pneumothorax (i). Magnification 250x.

Lung cancer resections had higher expression levels of TIMP-1 compared to pneumothorax and transplant biopsies while the level of KDR was higher in both the lung cancer and transplant biopsies. The levels of TIMP-2 were higher in the transplant group only. No differences were found in PGF, FLT1, or MMP-9 expression. The expression of VEGFA and MMP-2 was lower in lung cancer resections compared to pneumothorax and the transplant biopsy. Representative images of bronchial staining can be seen in Figures 3(a), 3(b), and 3(c).

#### 3.3.2. Alveolar Lining

All groups had similar expression levels for most of the proteins analysed except TIMP-1 which was higher in the lung cancer resection, MMP-2 which was higher in lung transplant biopsy and VEGFA, which was elevated in pneumothorax samples. Representative images of alveolar staining can be seen in Figures 3(d), 3(e), and 3(f).

#### 3.3.3. Macrophages

There was weaker staining of MMP-2 and VEGFA in the transplant biopsies, and the levels of PGF were found to be lower in lung cancer resections. Representative images of macrophage staining can be seen in Figures 3(g), 3(h), and 3(i).

The results of the scoring of the immunoreactivity for all of the antibodies studied are shown in Table 3.

Based on the results, we established a list of biomarkers that are unsuitable for gene expression and immunohistochemical studies (Table 4). These markers showed altered expression in lung cancer, transplant biopsies, or pneumothorax samples.

## 4. Discussion

Obtaining control tissue from completely well patients is difficult. As a result specimens from “nonnormal” sources are used frequently as controls in human research. This study aimed to assess three different sources of control lung. We compared the gene and protein expression of several lung injury biomarkers important in lung remodelling in cancer resections, biopsies from stable lung transplantation patients, and pneumothorax specimens. Samples from cancer resections and biopsies from stable lung transplantation were obtained after surgery and separated into two tubes with RNA*later* for subsequent RNA extraction and formalin for histological analysis. The pneumothorax samples were obtained from archived formalin-fixed paraffin-embedded tissue blocks. Intact RNA was successfully extracted from only 7 of the 15 pneumothorax samples. The number of samples would need to be increased in future studies to compensate for the possible failure of RNA extraction methods. The age of paraffin blocks is also one of the main determining factors of RNA quality, so future extraction would be better focussed on younger tissue blocks. These results should, therefore, be interpreted with caution as the quality of mRNA extracted is often poor.

The gene expression level of VEGFA, PGF, and their receptors FLT1 and KDR as well as MMP-2 and the inhibitors TIMP-1 and TIMP-2 was higher in samples from lung cancer resections compared to pneumothorax and transplant biopsy samples. Immunohistochemical studies showed lower expression levels of VEGFA and MMP-2 in lung cancer resections compared to pneumothorax and transplant biopsies, while in contrast TIMP-1 and KDR expression was higher in lung cancer. This finding is the opposite to previously published findings showing significantly lower gene and protein expression of all VEGF isoforms in control samples compared to ARDS in a murine model [22] in which the control group was derived from carefully selected normal lung.

Despite the high gene expression levels of VEGFA, PGF, FLT1, KDR, MMP-2, TIMP-1, and TIMP-2, only KDR and TIMP-1 were high at the protein level in the lung cancer resections. This could be due to the presence of abundant macrophages and PMNs which might contribute to the higher total gene expression level. A second possible explanation is that these genes are not regulated at a transcriptional level. There are many studies showing discrepancies between gene and protein expression which can be attributed to differences in gene regulatory mechanisms [23–26]. Posttranscriptional processes can be affected by mRNA stability and changes in translational efficiency. Some mRNAs are strongly retained in the nucleus, which can lead to their levels being overestimated relative to protein levels [23]. Gene expression is also representative of the entire lung while protein expression was assessed separately in the bronchial and alveolar lining and in inflammatory cell infiltrate.

VEGFA is known to be upregulated in several forms of human cancer [11, 27] and is involved in multiple pathways including remodelling in normal and pathological conditions. The high gene expression in lung cancer specimens is not unexpected and could be due to an angiogenic response to the nearby tumour. VEGFA binds both the FLT1 and KDR receptors though the majority of its effects are mediated through KDR [28]; hence, an increase in the expression of VEGFA is likely to be associated with an increase in both FLT1 and KDR. PGF mediates most of its effects through the FLT1 receptor and is expressed in the placenta, heart, and lungs. It has been shown that neutralising PGF can reduce the infiltration of angiogenic macrophages in tumours [29] so it may be an important target in cancer. PGF levels have been shown to be increased in several forms of cancer including non-small-cell lung cancer, correlating with tumour stage [30]. MMP-2 is also upregulated in pathological conditions including cancer [18, 19] but like VEGFA is also elevated in normal processes including embryonic development and tissue remodelling so an increase in lung cancer specimens could be expected [20, 21]. TIMP-2 binds to the hemopexin- like domain of pro-MMP-2, and, therefore, an elevation in MMP-2 is likely to be followed by a corresponding increase in the levels of its inhibitor. Interestingly the gene expression levels of MMP-9 were decreased in lung cancer specimens. MMP-9 has a more specific role in pathological remodeling, and gene regulation may be specific to the tumour region. TIMP-1 was higher at both the transcriptional and protein levels in the lung cancer specimens. TIMP-1 complexes with metalloproteinases by binding to their catalytic zinc cofactor and is known to act on numerous forms of MMP.

Analysis of the lung tissue from the four nonsmoking patients showed no significant difference to the smoking group (data not shown) even though lung cancer in nonsmokers is different on a genetic, cellular, and molecular level [31]. This suggests that the gene expression changes analysed in this study relate to the tumour, not smoking damage per se.

Our data shows that researchers need to exercise caution in choosing which group they use as a source of control tissue. The results of this study raise questions about the validity of using lung cancer resections as controls. In particular its use as a control for genes involved in development, remodeling, and pathological conditions is inappropriate. There is, however, the possibility that lung cancer resection tissue could be used in a limited way for immunohistochemical studies particularly if the assessment involves alveolar lining and macrophage infiltrate.

Our study shows that pneumothorax samples and lung tissue from stable lung transplant patients can be used as an alternative to lung cancer resections and is likely to be a better representative. MMP-2 and VEGFA in the alveolar area and macrophage infiltrate and KDR and TIMP-2 in the bronchial lining of transplant biopsies are the only exceptions. Archived formalin-fixed paraffin-embedded tissue is an alternative source provided that the RNA integrity is maintained, which may require increased numbers of samples to undergo RNA extraction. Our results highlight the need for researchers to be careful in selecting appropriate controls.

## Figures and Tables

**Table 1:** Primer information for Real time PCR.

Gene	Full gene name	ABI assay ID	Category
VEGFA	Vascular endothelial growth factor A	Hs00173626_m1	Growth factor
PGF	Placental growth factor	Hs01119262_m1	Signal transduction
FLT1	Fms-related tyrosine kinase 1	Hs01052945_m1	Signal transduction
KDR	Kinase insert domain receptor	Hs00911692_m1	Cell proliferation and differentiation
MMP-2	Matrix metalloproteinase 2	Hs00234422_m1	Calcium binding
MMP-9	Matrix metalloproteinase 9	Hs00234579_m1	Calcium binding
TIMP-1	Tissue inhibitor of matrix metalloproteinase 1	Hs00171558_m1	Protein metabolism and modification
TIMP-2	Tissue inhibitor of matrix metalloproteinase 2	Hs01091319_m1	Protein metabolism and modification
GUSB	Beta-glucuronidase	4333767T	Carbohydrate metabolism
HGPRT	Hypoxanthine phosphoribosyltransferase	4333768T	Nucleoside, nucleotide, and nucleic acid metabolism
B2M	Beta-2-microglobulin	4333766T	Immunity and defence

**Table 2:** Patient demographics.

	Cancer	Transplant	Pneumothorax
Age	37–81 (median 68) years	22–61 (median 40) years	17–65 (median 24) years

Gender	14 females 15 males	13 females 10 males	3 females 4 males

Diagnosis	Adenocarcinoma (*n* = 15) Squamous cell carcinoma (*n* = 5) Carcinoid tumour (*n* = 3) Large cell carcinoma (*n* = 3) Intralobular pulmonary sequestration (*n* = 1) Usual interstitial pneumonia (*n* = 1) Cryptococcoma (*n* = 1)	Bilateral lung transplant	Pneumothorax

Smoking history	Yes (*n* = 25) No (*n* = 4)	Yes (*n* = 1) No (*n* = 25)	No (*n* = 7)

**Table 3:** Immunoreactivity scores for antibodies in lung derived from cancer resection, transplant biopsy, and pneumothorax.

Antibody	Cancer			Transplant			Pneumothorax		
	BL	AL	MPG	BL	AL	MPG	BL	AL	MPG
VEGFA	0-1	0-1	2	3	0	1	3	2	3
PGF	0-1	0	0	0-1	0	2	0-1	0	2
FLT1	0-1	0-1	2	0-1	0-1	2	0-1	0-1	2
KDR	3	1	3	3	1	2	0-1	0-1	2
MMP-2	2	0-1	2	3	3	1	3	0	3
MMP-9	0-1	0	1	0-1	0	0	0-1	0	1
TIMP-1	2-3	2	2	0-1	1	2	0-1	0	2
TIMP-2	0-1	0	1	2	1	2	0-1	0	2

BL: bronchiole lining.

AL: alveolar lining.

MPG: macrophages.

**Table 4:** Biomarkers unsuitable for gene and protein expression studies in cancer resection, transplant biopsy, and pneumothorax.

	Cancer resection	Transplant biopsy	Pneumothorax
Gene expression	VEGFA, PGF, FLT1, KDR, MMP-2, MMP-9, TIMP-1, TIMP-2	None	None
Protein expression			
Bronchial lining	VEGFA, TIMP-1, KDR, MMP-2	KDR, TIMP-2	None
Alveolar lining	TIMP-1	MMP-2	VEGFA
Macrophages	PGF	MMP-2, VEGFA	VEGFA

## References

[1] Andersson-Sjöland A, Erjefält JS, Bjermer L, Eriksson L, Westergren-Thorsson G (2009). Fibrocytes are associated with vascular and parenchymal remodelling in patients with obliterative bronchiolitis. *Respiratory Research*.

[2] Baraldo S, Turato G, Badin C (2004). Neutrophilic infiltration within the airway smooth muscle in patients with COPD. *Thorax*.

[3] Charpin JM, Stern M, Lebrun G, Aubin P, Grenet D, Israël-Biet D (2001). Increased endothelin-1 associated with bacterial infection in lung transplant recipients. *Transplantation*.

[4] Koli K, Myllärniemi M, Vuorinen K (2006). Bone morphogenetic protein-4 inhibitor Gremlin is overexpressed in idiopathic pulmonary fibrosis. *American Journal of Pathology*.

[5] McLaren J, Prentice A, Charnock-Jones DS (1996). Vascular endothelial growth factor is produced by peritoneal fluid macrophages in endometriosis and is regulated by ovarian steroids. *Journal of Clinical Investigation*.

[6] Sugishita Y, Shimizu T, Yao A (2000). Lipopolysaccharide augments expression and secretion of vascular endothelial growth factor in rat ventricular myocytes. *Biochemical and Biophysical Research Communications*.

[7] Lahm T, Crisostomo PR, Markel TA, Wang M, Lillemoe KD, Meldrum DR (2007). The critical role of vascular endothelial growth factor in pulmonary vascular remodeling after lung injury. *Shock*.

[8] Kaner RJ, Ladetto JV, Singh R, Fukuda N, Matthay MA, Crystal RG (2000). Lung overexpression of the vascular endothelial growth factor gene induces pulmonary edema. *American Journal of Respiratory Cell and Molecular Biology*.

[9] Tsao PN, Su YN, Li H (2004). Overexpression of placenta growth factor contributes to the pathogenesis of pulmonary emphysema. *American Journal of Respiratory and Critical Care Medicine*.

[10] Krenn K, Klepetko W, Taghavi S, Lang G, Schneider B, Aharinejad S (2007). Recipient vascular endothelial growth factor serum levels predict primary lung graft dysfunction. *American Journal of Transplantation*.

[11] Yang CC, Chu KC, Yeh WM (2004). The expression of vascular endothelial growth factor in transitional cell carcinoma of urinary bladder is correlated with cancer progression. *Urologic Oncology*.

[12] Granata F, Frattini A, Loffredo S (2010). Production of vascular endothelial growth factors from human lung macrophages induced by group IIA and group X secreted phospholipases A2. *Journal of Immunology*.

[13] Yang W, Ahn H, Hinrichs M, Torry RJ, Torry DS (2003). Evidence of a novel isoform of placenta growth factor (P1GF-4) expressed in human trophoblast and endothelial cells. *Journal of Reproductive Immunology*.

[14] Chen CN, Hsieh FJ, Cheng YM (2004). The significance of placenta growth factor in angiogenesis and clinical outcome of human gastric cancer. *Cancer Letters*.

[15] Luttun A, Tjwa M, Moons L (2002). Revascularization of ischemic tissues by PLGF treatment, and inhibition of tumor angiogenesis, arthritis and atherosclerosis by anti-Flt1. *Nature Medicine*.

[16] Thickett DR, Armstrong L, Christie SJ, Millar AB (2001). Vascular endothelial growth factor may contribute to increased vascular permeability in acute respiratory distress syndrome. *American Journal of Respiratory and Critical Care Medicine*.

[17] Shimoyama T, Tabuchi N, Chung J, Koyama T, Sunamori M (2006). Matrix metalloproteinase inhibitor (ONO-4817) attenuates ischemia-reperfusion injury in rat lung. *Medical Science Monitor*.

[18] Chow AK, Cena J, Schulz R (2007). Acute actions and novel targets of matrix metalloproteinases in the heart and vasculature. *British Journal of Pharmacology*.

[19] Rydlova M, Holubec L, Ludvikova M (2008). Biological activity and clinical implications of the matrix metalloproteinases. *Anticancer Research*.

[20] Gill SE, Parks WC (2008). Metalloproteinases and their inhibitors: regulators of wound healing. *International Journal of Biochemistry and Cell Biology*.

[21] Page-McCaw A, Ewald AJ, Werb Z (2007). Matrix metalloproteinases and the regulation of tissue remodelling. *Nature Reviews Molecular Cell Biology*.

[22] Medford AR, Douglas SK, Godinho SI (2009). Vascular Endothelial Growth Factor (VEGF) isoform expression and activity in human and murine lung injury. *Respiratory Research*.

[23] Gry M, Rimini R, Strömberg S (2009). Correlations between RNA and protein expression profiles in 23 human cell lines. *BMC Genomics*.

[24] Kerst G, Bergold N, Gieseke F (2008). WT1 protein expression in childhood acute leukemia. *American Journal of Hematology*.

[25] Kudo C, Ajioka I, Hirata Y, Nakajima K (2005). Expression profiles of EphA3 at both the RNA and protein level in the developing mammalian forebrain. *Journal of Comparative Neurology*.

[26] Tank AW, Xu L, Chen X, Radcliffe P, Sterling CR (2008). Post-transcriptional regulation of tyrosine hydroxylase expression in adrenal medulla and brain. *Annals of the New York Academy of Sciences*.

[27] Takekoshi K, Isobe K, Yashiro T (2004). Expression of vascular endothelial growth factor (VEGF) and its cognate receptors in human pheochromocytomas. *Life Sciences*.

[28] Matsumoto T, Claesson-Welsh L (2001). VEGF receptor signal transduction. *Sci STKE*.

[29] Fischer C, Jonckx B, Mazzone M (2007). Anti-PlGF inhibits growth of VEGF(R)-inhibitor-resistant tumors without affecting healthy vessels. *Cell*.

[30] Zhang L, Chen J, Ke Y, Mansel RE, Jiang WG (2005). Expression of placenta growth factor (PIGF) in non-small cell lung cancer (NSCLC) and the clinical and prognostic significance. *World Journal of Surgical Oncology*.

[31] Rudin CM, Avila-Tang E, Harris CC (2009). Lung cancer in never smokers: molecular profiles and therapeutic implications. *Clinical Cancer Research*.

